# Editorial: Impact of environmental factors on the health of children and older adults

**DOI:** 10.3389/fpubh.2025.1679201

**Published:** 2025-11-14

**Authors:** Yichen Wang, Jiao Lu, Xin Long, Zhaoling Li, Jiayuan Wang

**Affiliations:** 1 School of Public Policy and Administration, Xi'an Jiaotong University, Xi'an, Shaanxi, China; 2 Research Centre for Atmospheric Environment, Chongqing Institute of Green and Intelligent Technology, Chinese Academy of Sciences, Chongqing, China; 3 School of Economics, Shanghai University, Shanghai, China; 4 Air Quality Research Center, University of California, Davis, Davis, CA, United States

**Keywords:** environmental factors, health, children, older adults, exposures

Environmental exposures are among the most pressing public health challenges today. Vulnerable groups, particularly children and older adults, bear a disproportionate burden of these exposures. This Research Topic, “*Impact of environmental factors on the health of children and older adults*,” brought together 14 original studies to address the multifaceted health effects of air pollution, toxic exposures, and related environmental risks on these sensitive populations.

## Scope and relevance

Air pollution and environmental pollutants have been implicated in a broad range of health conditions including asthma, developmental disorders, cardiovascular disease, and premature mortality. As climate change accelerates, these risks are likely to increase, exacerbating health disparities. This Topic focused on quantifying these effects, developing exposure models, and advancing tools to support public health interventions, particularly in the context of children's development and older adults' chronic conditions.

## Article highlights

This Research Topic presents a collection of studies that explore the impact of environmental factors on children's health. Li et al. conducted a nationwide study involving 32,000 children in China and found that 4.1% had elevated blood lead levels. Risk was significantly higher among children exposed to poor air quality and secondhand smoke (Li et al.). Bao et al. analyzed time-series data on pediatric asthma hospitalizations in Eastern China. Although seasonal trends were evident, no consistent association was found with air pollution, suggesting other mediating factors may influence hospitalization risk (Bao et al.). In Qatar, Husein et al. assessed how meteorological conditions affect the transmission of viral respiratory infections. They identified school-aged children as key drivers of seasonal outbreaks, with temperature and humidity significantly shaping transmission patterns (Husein et al.).

In relation to older adults, Zhang et al. shows that long-term exposure to PM_2.5_ chemical components significantly increases the risk of metabolic syndrome and its components—particularly central obesity, high blood pressure, high fasting glucose, and low HDL—with stronger effects observed among single, divorced, or widowed individuals. In relation to older adults, He et al. estimated the global ischemic heart disease (IHD) burden attributable to particulate matter (PM) pollution in populations aged 70 and above. While global age-standardized DALY rates have declined, total DALYs are projected to rise through 2044 due to aging populations, particularly in low- and middle-income countries (He et al.).

The Topic also includes several studies on environmental risk modeling and assessment. Yang et al. used machine learning to predict outpatient visits for respiratory illness, identifying ozone concentration and temperature as the most influential predictors. Kobza et al. analyzed ozone levels in Upper Silesia, Poland, finding a complex dual effect where low NOx concentrations led to increased ozone formation.

Behavioral and community health dimensions are also addressed. Kaplan et al. developed and validated a scale to measure public awareness of BPA (bisphenol A) exposure, offering a practical tool for future behavioral research. Cervantes et al. reviewed that fungal contamination in schools poses a serious threat to indoor air quality and student health, is influenced by geographic location and season, and requires targeted monitoring and control supported by diverse sampling methods, molecular analysis, and standardized data.

Regarding regional and dietary exposures, Wu et al. demonstrates that pulmonary embolism patients at extremely high altitudes exhibit distinct hematological abnormalities, faster thrombus resolution, and heightened susceptibility among younger individuals, with abnormal uric acid metabolism emerging as a potential risk factor (Wu et al.).

Finally, studies also examined household, occupational and urban environmental risks. Tan et al. finds that household use of solid fuels for cooking or heating significantly increases the risk of cognitive frailty, while switching to clean fuels may reduce this risk. Toure et al. shows that while communities in Siguiri's artisanal mining areas demonstrate moderate knowledge, positive attitudes, and largely adequate practices regarding water pollution, critical gaps in risk awareness highlight the need for integrated education, awareness, and technical support to foster sustainable behaviors. Zhang et al. suggests that insecticide exposure is associated with cognitive impairment in older adults, particularly affecting memory and delayed recall, though further longitudinal research is needed to confirm causality.

## Conclusion

Collectively, the 14 articles in this Research Topic offer a rich and multidisciplinary perspective on how environmental exposures affect the health of children and older adults. From novel exposure assessment tools and AI models to global burden estimates and local risk perception studies, this Research Topic underscores the urgency of tailored environmental health policies for vulnerable populations.

We extend our gratitude to the authors, reviewers, and editorial staff for contributing to this important endeavor. We hope the findings here inform future environmental health governance, supporting healthier environments across all stages of life.

